# Low Blood Long Chain Omega-3 Fatty Acids in UK Children Are Associated with Poor Cognitive Performance and Behavior: A Cross-Sectional Analysis from the DOLAB Study

**DOI:** 10.1371/journal.pone.0066697

**Published:** 2013-06-24

**Authors:** Paul Montgomery, Jennifer R. Burton, Richard P. Sewell, Thees F. Spreckelsen, Alexandra J. Richardson

**Affiliations:** Centre for Evidence-Based Intervention, University of Oxford, Oxford, United Kingdom; University of California, San Francisco, United States of America

## Abstract

**Background:**

Omega-3 long-chain polyunsaturated fatty acids (LC-PUFA), especially DHA (docosahexaenonic acid) are essential for brain development and physical health. Low blood Omega-3 LC-PUFA have been reported in children with ADHD and related behavior/learning difficulties, as have benefits from dietary supplementation. Little is known, however, about blood fatty acid status in the general child population. We therefore investigated this in relation to age-standardized measures of behavior and cognition in a representative sample of children from mainstream schools.

**Participants:**

493 schoolchildren aged 7–9 years from mainstream Oxfordshire schools, selected for below average reading performance in national assessments at age seven.

**Method:**

Whole blood fatty acids were obtained via fingerstick samples. Reading and working memory were assessed using the British Ability Scales (II). Behaviour (ADHD-type symptoms) was rated using the revised Conners’ rating scales (long parent and teacher versions). Associations were examined and adjusted for relevant demographic variables.

**Results:**

DHA and eicosapentaenoic acid (EPA), accounted for only 1.9% and 0.55% respectively of total blood fatty acids, with DHA showing more individual variation. Controlling for sex and socio-economic status, lower DHA concentrations were associated with poorer reading ability (std. OLS coeff. = 0.09, p = <.042) and working memory performance (0.14, p = <.001). Lower DHA was also associated with higher levels of parent rated oppositional behavior and emotional lability (−0.175, p = <.0001 and −0.178, p = <.0001).

**Conclusions:**

In these healthy UK children with below average reading ability, concentrations of DHA and other Omega-3 LC-PUFA were low relative to adult cardiovascular health recommendations, and directly related to measures of cognition and behavior. These findings require confirmation, but suggest that the benefits from dietary supplementation with Omega-3 LC-PUFA found for ADHD, Dyspraxia, Dyslexia, and related conditions might extend to the general school population.

## Introduction

### Background and Rationale

Omega-3 long-chain polyunsaturated fatty acids (LC-PUFA) play key roles in normal brain development and functioning as well as in cardiovascular and immune system health [1], [2]. Omega-3 are dietary essentials, but average intakes are low in most modern developed countries relative to Omega-6 LC-PUFA [3]. Increasing evidence indicates that this imbalance may be contributing to a wide range of physical and mental health disorders [4]. These include common childhood behavior and learning difficulties such as ADHD, dyslexia, dyspraxia and related conditions [5], [6].

Low blood Omega-3 LC-PUFA concentrations have been reported in children with ADHD and related behavior or learning difficulties [7], [8] as have benefits from dietary supplementation [9]–[11]. Furthermore, in one study of boys with and without ADHD, links between low blood Omega-3 status and behavior as well as general health problems were found to extend across the combined sample [7], suggesting that a lack of these fatty acids may also have important consequences in the general population [12]. Similarly, low Omega-3 levels were associated with reading performance in a sample of dyslexic and non-dyslexic adults [13].

Currently, however, little is known about the LC-PUFA status of UK children – mainly because until recently, this could only be assessed using venous blood samples. Only one published study has attempted to explore LC-PUFA status in mainstream UK school children in relation to cognitive and behavioral measures [14]. However, the study used a buccal cell method previously untested in children, rather than well-validated blood measures. In recent years, methods of assessing blood fatty acid status from a fingerstick sample of whole blood have been developed and validated [15], [16]. This has made it ethically possible to investigate blood Omega-3 concentrations in healthy children from the general UK population.

### Objectives

The aims of this study were twofold: First, to address the lack of evidence regarding the LC-PUFA status of UK children; by way of examining the distribution of whole blood fatty acid concentrations, in a representative sample of healthy children from mainstream schools. Second, to expand current knowledge regarding the relevance of LC-PUFA for cognition and behavior; by investigating associations between blood Omega-3 and Omega-6 and children’s reading, working memory and ADHD-type symptoms.

Our prediction was that lower blood Omega-3 and Omega-6 LC-PUFA would be associated with poorer behavior and cognitive performance. This follows from:

1. the physiological importance of LC-PUFA to brain function;
2. existing research documenting low LC-PUFA in clinically defined behavior and learning difficulties;
3. emerging evidence of benefits from supplementation from LC-PUFA for child behavior and learning (as noted above).

## Methods

### Study Design

This is a cross-sectional, observational study, involving children from mainstream primary schools in Oxfordshire, a large county in the UK. It formed the screening stage of an intervention study to determine whether supplementation with the Omega-3 fatty acid DHA would improve reading, working memory and behavior (The DOLAB study) [17]. For this reason the sampling was based on the children’s performance in reading according to local authority data.

### Population

Invitations to participate were issued to 1376 healthy children from year groups 3, 4 and 5 (mostly aged 7–9 years). Recruitment of schools took place in collaboration with Oxfordshire local authority between January 2009 and November 2010 and 74 schools participated. Invitation packs were sent to parents/guardians of all children who met entry criteria.

Children were eligible if they had no significant learning difficulty (i.e. they were not statemented as having “Special Educational Needs” [18]); their first language at home was English; and their reading ability was below average according to national assessments at age 7 years [19] and/or their teachers’ current judgment. Children were not excluded if they had previously been diagnosed with ADHD and/or dyslexia.

In order to minimize potential sources of bias, attempts were made to involve as wide a range of schools as possible (e.g. in terms of size and type of location) whilst also ensuring that the overall percentage of children from low-income groups was similar to the national average. Details of participation were recorded at school, parent and child level to ensure that potential sampling bias could be taken into account. Further, every effort was made to telephone all parents to explain the purpose of the study and maximize recruitment. Well-validated, standardized tests were used to minimize measurement bias.

### Ethics

Written informed consent was gained from parents, and verbal assent from the children, prior to the initial screening assessments. Ethical consent was gained from the Milton Keynes NHS Research Ethics Committee (08/H0603/49). Project approval was gained from the Oxfordshire local authority to conduct research in schools.

### Variables

#### Demographics

Information on eligibility for free school meals (FSM) was obtained from Local Authority data and used as a proxy for Social Economic Status (SES) [20]. Local Authority data were also used to report sex and age. Additional information was gathered from parents on children’s current consumption of fish, use of medication and use of Omega-3 supplements. (See Materials S1).

#### Methods for Capillary Whole Blood Fatty Acid Analysis

Drops of capillary whole blood were collected onto filter paper using a lancet device applied to the child’s finger. Samples were analyzed for the fatty acid composition of total lipids via gas-liquid chromatography using a well-validated protocol [15], [21]. Individual fatty acids were expressed as a percent of the total µg of fatty acid (weight percent). (See Materials S2). Data are reported only for Omega-3 and Omega-6 LC-PUFA as these are the focus of this study. Furthermore the sum of DHA and EPA is reported throughout this paper as broadly equating to the “Omega-3 Index” [22], [23]. This measure was developed as an index of cardiovascular risk and is conventionally assessed as DHA+EPA in red blood cell membranes. The results here are obtained from whole blood, and therefore should only be compared cautiously with “Omega-3 Index” as reported elsewhere.

#### Reading

Reading was assessed using the Word Reading Achievement sub-test of the British Ability Scales 2^nd^ Edition (BAS II) [24]. This is an age-standardized, single word reading test, normed on UK children, with a mean of 100 and a standard deviation of 15.

#### Working Memory

Working memory was assessed using the Recall of Digits Forward and Recall of Digits Backward sub-tests from the BAS II. Again, these measures are age standardized, but use T-scores, with a mean of 50 and a standard deviation of 10.

#### Behavior

ADHD-type symptoms were assessed by both parents and teachers using long versions of the Conners’ Rating Scales (CPRS-L and CTRS-L) [25]. These are age and gender-standardized, highly valid and reliable scales measuring child behavior over several domains and are expressed as T-scores (mean = 50, sd = 10).

#### Statistical Methods

For descriptive statistics, means and standard deviations were calculated for reading, working memory, behavior (ADHD-type symptoms) and Omega-3 and Omega-6 PUFA fatty acid values from the capillary whole blood analyses. Comparisons were performed using Mann-Whitney and Kruskal-Wallis tests accounting for the distributional nature of the data. Correlations were examined using Spearman’s correlation coefficients. Adjusted coefficients were estimated using ordinary linear regressions (OLS). Corrections for multiple comparisons were not considered appropriate because scores on many of the measures used are intercorrelated. This is particularly true of the behavior ratings, as the individual Conners’ scales are derived from different but overlapping combinations of the items in these inventories. Raw coefficients are presented alongside coefficients standardized using the ratio of standard deviations (sd_x_/sd_y_). All analyses were conducted using Stata MP Version 11.2.

## Results

### Participants

**Figure 1:**
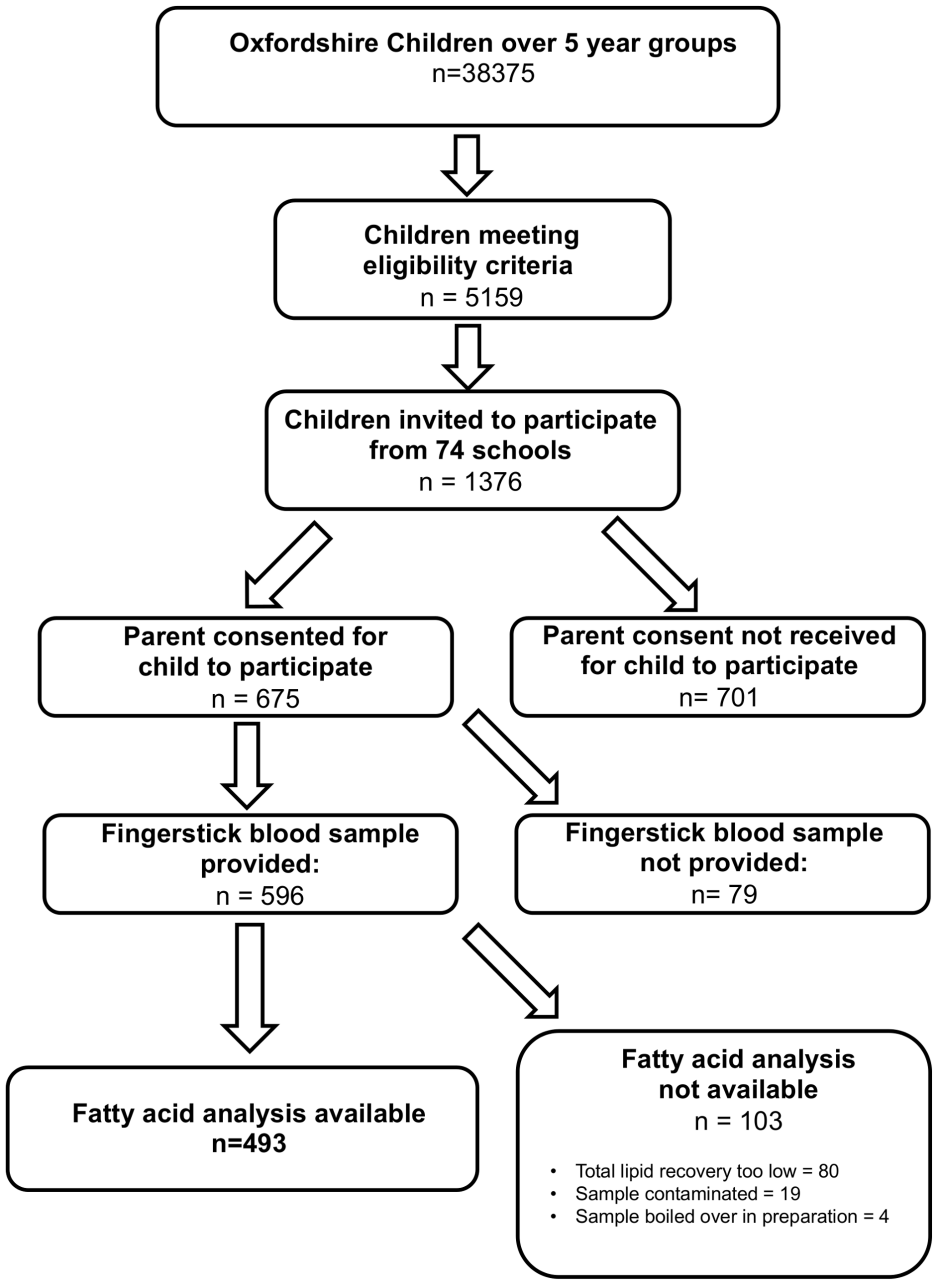
Flowchart of participants on whom blood data available.

Over five thousand children from a cohort of 38,375 attending 234 Oxfordshire primary schools met inclusion criteria over the 23 months of recruitment into the study. From the 74 volunteering schools, 1376 children were invited to take part, and 675 children whose parents/guardians consented were assessed. Five hundred and ninety-six (88%) of these children agreed to provide a fingerstick blood sample. Of these 103 (17%) blood samples were either too small, or contaminated or “boiled over” during the chemical analysis. Not all children could provide sufficient blood for reliable analysis. Contamination can arise from the reagents used to dissolve fatty acids from the filter paper. Occasionally, samples can be lost through “boiling over” during the heating process required. (See Materials S1, for details of the analysis procedure.) - In total 493 samples were available for which fatty acid data could be analyzed for this paper (see Flowchart, Figure 1).

### Descriptive Data

Children who accepted the invitation to take part in the study (n = 675) did not differ significantly from those who declined (n = 701) in terms of age (χ^2^ = .181 (df = 2, n = 1376), p<.91), sex (χ^2^ = 1.58 (df = 1, n = 1376), p<.21) or eligibility for Free School Meals (FSM) (χ^2^ = 1.11 (df = 1, n = 1376), p<.29). Data on ethnicity for children who did not participate were not available.

Participants for whom blood fatty acid data were available did not differ significantly from those without these data in terms of age (χ_2_ = .568 (df = 2, n = 675), p<.75) or sex (χ_2_ = .209 (df = 1, n = 675), p<.65); although blood data were available for fewer children in receipt of free school meals: (χ_2_ = 3.75 (df = 1, n = 675), p<.053). Comparisons of ethnicity were not meaningful since our sample was overwhelmingly white.

Information on all participants (n = 675), on the children who had blood taken (n = 596), and those for whom blood fatty acid data were available (n = 493) are shown in Table 1.

**Table 1:** Participant characteristics† and blood data availability.

		Blood data available	Blood taken	Total sample
		(n = 493)	(n = 596)	(n = 675)
**Sex, n (%)**				
	Female	221 (44.8%)	256 (43.0%)	299 (44.3%)
	Male	272 (55.2%)	340 (57.1%)	376 (55.7%)
**Age in years, n (%)**				
	6/7 years	136 (27.6%)	157 (26.3%)	183 (27.1%)
	8 years	177 (35.9%)	215 (36.1%)	248 (36.7%)
	9/10 years	180 (36.5%)	224 (37.6%)	244 (36.1%)
**Free School Meals, n (%)**				
	Not eligible	416 (84.4%)	500 (83.9%)	558 (82.7%)
	Eligible	77 (15.6%)	96 (16.1%)	117 (17.3%)
**Ethnicity, n (%)**				
	White	453 (91.9%)	543 (91.1%)	615 (91.1%)
	Mixed	17 (3.4%)	9 (1.5%)	10 (1.5%)
	Asian	7 (1.4%)	3 (0.5%)	4 (0.6%)
	Other	5 (1.0%)	22 (3.7%)	23 (3.4%)
	Black	1 (0.2%)	8 (1.3%)	9 (1.3%)
	Unknown	10 (2.0%)	11 (1.8%)	14 (2.1%)
**Dyslexia diagnosis, n (%)**				
	No	443 (89.9%)	532 (89.3%)	603 (89.3%)
	Yes	31 (6.3%)	41 (6.9%)	47 (7.0%)
**ADHD diagnosis, n (%)**				
	No	451 (91.5%)	547 (91.8%)	621 (92.0%)
	Yes	5 (1.0%)	6 (1.0%)	6 (0.9%)

† Demographic data provided by the Oxfordshire Local Authority.

### Outcome Variables

The mean levels of reading ability and working memory (forward/backward) in the sample were 90.58 (sd = 10.55) and 41.75 (sd = 8.02)/45.15 (sd. = 6.83) respectively, i.e. within the normal population ranges. Mean behavior scores (ADHD-type symptoms) were also within the normal population range for all subscales and global scales (See Table S1).

**Figure 2:**
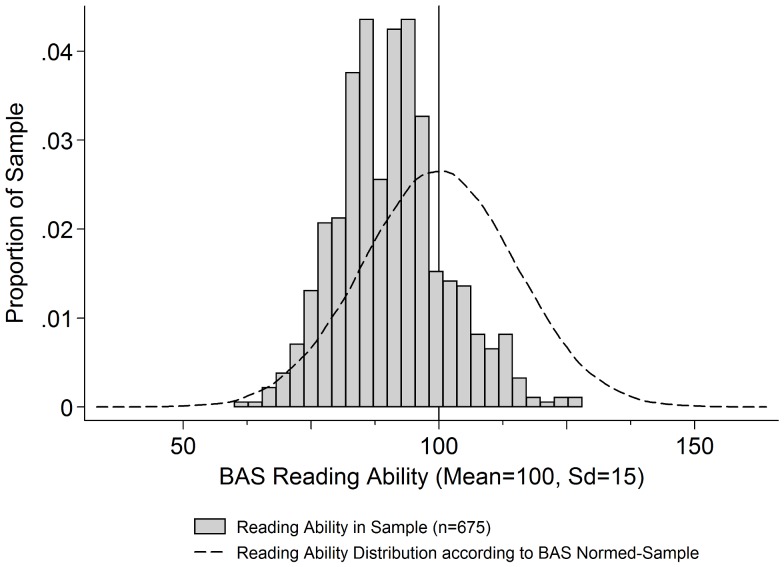
Reading ability in the study sample versus normed sample school children. Note: Normed distribution based on random draws from a normal distribution (mean = 100, sd = 15).

Comparing BAS II reading ability of the present sample with a normed distribution for the UK shows that the present sample has a slightly lower reading ability level (mean = 90.58, sd = 10.55) whilst maintaining a roughly similar distribution shape (see Figure 2).

### Main Results– Fatty Acid Analysis

#### Fatty Acids

Associations between blood fatty acid concentrations and both demographic characteristics and cognitive function were examined for all children whose blood data were available (see Table S2).

**Figure 3:**
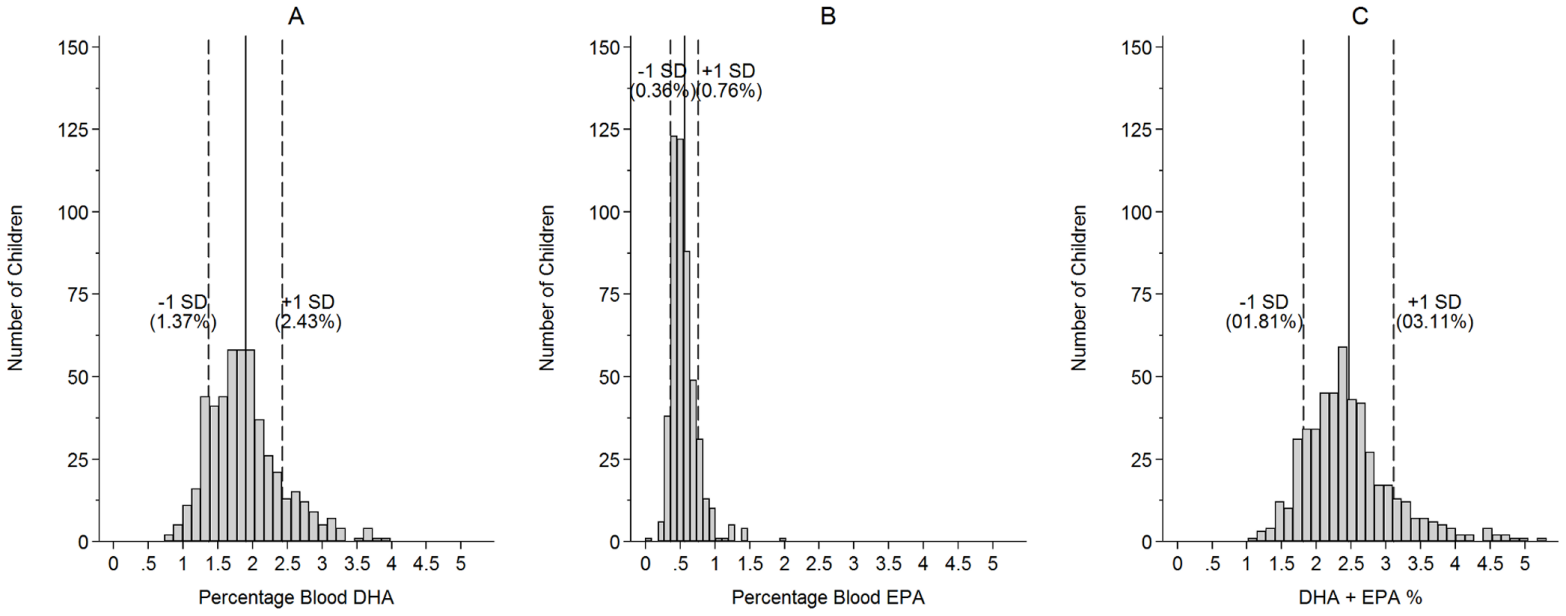
Distribution of DHA, EPA, and the“EPA+DHA (“Omega-3 Index”)” in school children. A: Docosahexaenoic acid (22∶6, n-3) B: Eicosapentaenoic acid(20∶5, n-3) C: DHA+EPA% (Omega-3 Index) Data: n = 493.

Focusing on the Omega-3 LC-PUFA central to cognition and behavior (DHA and EPA), DHA varied substantially more across children than EPA (DHA, mean = 1.90% sd = 0.53%; EPA, mean = 0.55%, sd = 0.26%) (see Figure 3).

The Omega-3 Index, DHA and EPA combined, had a mean of 2.46% (sd = 0.65%) (see Figure 3).

#### Demographic Information and Fatty Acid Status

Blood fatty acid concentrations were compared for groups defined by sex, age and socioeconomic status, and these data are reported in Table 2. (Significance-test results are reported in Table S3).

**Table 2:** Capillary whole blood fatty acid concentrations (% of total lipids, means and sds) by demographic characteristics† and tests for significant differences between groups within each demographic characteristic.

	Total		Gender		Age		Socio-Economic-Status	
	mean (sd)	Male (sd)	Female (sd)	6/7years	8 years	9/10 years	No Freemeals (sd)	Freemeals (sd)
	(n = 493)	(n = 221)	(n = 272)	(n = 136)	(n = 177)	(n = 180)	(n = 416)	(n = 77)
*Omega-3 fatty acids*								
** ALA** (18∶3, n–3)	0.54 (0.254)	0.55 (0.255)	0.54 (0.252)	0.55 (0.31)	0.53 (0.212)	0.55 (0.246)	0.52 (0.323)	0.55 (0.239)
** SDA** (20∶3, n–3)	0.05 (0.065)	0.05 (0.063)	0.04 (0.066)	0.04 (0.056)	0.04 (0.052)	0.05 (0.08)	0.04 (0.06)	0.05 (0.066)
** EPA** (20∶5, n–3)	0.56 (0.200)	0.57 (0.218)	0.55 (0.175)	0.57 (0.245)	0.55 (0.174)	0.56 (0.186)	0.56 (0.183)	0.56 (0.203)
** DPA** (22∶5, n–3)	1.03 (0.266)	1.05 (0.185)	1.00 (0.339)^(m vs. f)^ ***	1.06 (0.218)	1.02 (0.311)	1.01 (0.25)^ (all ages)^ *	1.08 (0.408)	1.02 (0.230)
** DHA** (22∶6, n–3)	1.9 (0.53)	1.95 (0.53)	1.85 (0.525)	1.97 (0.568)	1.87 (0.471)	1.88 (0.551)	1.82 (0.454)	1.92 (0.541)
** EPA+DHA** (“Omega-3 Index”)	2.46 (0.651)	2.51 (0.658)	2.4 (0.639)	2.55 (0.734)	2.42 (0.55)	2.45 (0.673)	2.39 (0.532)	2.48 (0.671)
** Total Omega-3**	4.08 (0.823)	4.16 (0.813)	3.99 (0.828)^(m vs. f)^ ***	4.2 (0.935)	4.01 (0.731)	4.06 (0.813)	4.03 (0.766)	4.09 (0.834)
*Omega-6 fatty acids*								
** LA** (18∶2, n–6)	19.19 (2.294)	19.19 (2.356)	19.19 (2.220)	19.73 (2.064)	19.12 (2.378)	18.87 (2.314)^(all ages)^ **	19.45 (2.128)	19.15 (2.322)
** GLA** (18∶3, n–6)	0.31 (0.233)	0.34 (0.243)	0.28 (0.217)^(m vs. f)^ **	0.31 (0.228)	0.33 (0.251)	0.3 (0.22)	0.28 (0.22)	0.32 (0.235)
** DGLA** (20∶3, n–6)	1.56 (0.341)	1.62 (0.34)	1.48 (0.327)^(m vs. f)^ ***	1.55 (0.312)	1.56 (0.352)	1.56 (0.352)	1.5 (0.287)	1.57 (0.349)
** AA** (20∶4, n–6)	8.17 (1.31)	8.35 (1.262)	7.94 (1.335)^(m vs. f)^ ***	8.5 (1.329)	8.1 (1.224)	7.98 (1.337)^ (all ages)^ **	8.17 (1.395)	8.17 (1.296)
** Adrenic** (22∶4, n–6)	1.11 (0.221)	1.14 (0.215)	1.07 (0.221)^(m vs. f)^ ***	1.12 (0.223)	1.11 (0.221)	1.1 (0.219)	1.12 (0.24)	1.1 (0.217)
** DPA** (22∶5, n–6)	0.25 (0.100)	0.27 (0.100)	0.24 (0.096)w^(m vs. f)^ ***	0.26 (0.097)	0.26 (0.1)	0.25 (0.102)	0.27 (0.072)	0.25 (0.104)
** Total Omega-6**	30.84 (3.34)	31.17 (3.397)	30.44 (3.23)	31.71 (3.1)	30.71 (3.398)	30.32 (3.348)^ (all ages)^ **	31.06 (3.106)	30.8 (3.384)

**Asterisks** : Significance tests of simultaneous independence of all categories for one demographic characteristic:

* p<0.05;

** p<0.01;

*** p<0.001.

**Fatty Acid Acronyms:** ALA: “α-Linolenic acid”; SDA: “Stearidonic acid”; EPA: “Eicosapentaenoic acid”; DPA: “Docosapentaenoic acid” (both Omega-3 and Omega-6); DHA: “Docosahexaenoic acid”; LA: “Linoleic acid”; GLA: “Gamma-linolenic acid”; DGLA: “Dihomo-gamma-linolenic acid”; AA: “Arachidonic acid”.

† Demographic data provided by the Oxfordshire Local Authority.

Group comparisons by sex revealed significantly higher concentrations in males than females of two Omega-3 LC-PUFA, that is DHA (p<0.04) and DPA n-3 (p<0.001), and also EPA+DHA (“Omega-3 Index”) (p<0.023). Several Omega-6 fatty acids were also higher in males: DPA n-6 (p<0.03), Adrenic Acid, AA and DGLA (both p<.001), GLA (p<0.01) and Total Omega-6 (p<.02).

Children in the lowest age-group (7 years) showed higher concentrations of DPA (n-3), AA and LA (p<0.01) as well as Total Omega-6 (p<0.001). Similarly, age measured in months was significantly and inversely related to these fatty acids. (DPA (n–3) rho = −0.112 (p<0.013); AA rho = −0.122 (p<0.007); LA rho = −0.144 (p<0.001); Total Omega-6 rho = - 0.151 (p<0.001). None of the other fatty acids were found to be significantly related with a child’s age. No significant differences were found for socio-economic status as measured by eligibility for free school meals.

#### Fatty Acid Status and Fish Consumption

Parents reported that 432/488 (88.2%) children ate fish less than twice a week, and 9% did not eat fish at all.

Table 3 shows blood fatty acid concentrations in relation to children’s fish consumption. DHA, ALA, Total Omega-3 and the EPA+DHA (“Omega-3 Index”) were all significantly higher in children with higher fish consumption, while the Omega-6 fatty acids Adrenic, AA, and DGLA, were correspondingly lower.

**Table 3:** Blood fatty acid levels by fish consumption.

		Fish consumption					
		Not at all	Less than once a week	Once a week	More than once a week	?^2^ (P-value)†	
		(n = 44)	(n = 158)	(n = 232)	(n = 56)		
*Omega-3 fatty acids:*							
** ALA** (18∶3, n–3)		0.50	0.53	0.53	0.66	8.004 (0.046)	
** SDA** (20∶3, n–3)		0.06	0.05	0.04	0.05	2.562 (0.464)	
** EPA** (20∶5, n–3)		0.51	0.54	0.56	0.64	6.090 (0.107)	
** DPA** (22∶5, n–3)		1.00	1.03	1.05	0.95	7.138 (0.068)	
** DHA** (22∶6, n–3)		1.64	1.76	2.00	2.09	38.593 (0.000)	
** EPA+DHA** (“Omega-3 Index”)		2.15	2.30	2.56	2.73	33.003 (0.000)	
** Total Omega-3**		3.70	3.91	4.18	4.39	23.854 (0.000)	
*Omega-6 fatty acids:*							
** LA** (18∶2, n–6)		18.36	19.21	19.30	19.32	5.277 (0.153)	
** GLA** (18∶3, n–6)		0.30	0.34	0.31	0.26	1.964 (0.580)	
** DGLA** (20∶3, n–6)		1.58	1.58	1.56	1.45	8.873 (0.031)	
** AA** (20∶4, n–6)		8.32	8.13	8.27	7.71	7.882 (0.049)	
** Adrenic** (22∶4, n–6)		1.19	1.13	1.10	1.01	15.578 (0.001)	
** DPA** (22∶5, n–6)		0.26	0.26	0.26	0.24	3.755 (0.289)	
** Total Omega-6**		30.28	30.90	31.04	30.30	3.488 (0.322)	

† Kruskal-Wallis Test of simultaneous independence of all categories of fish consumption.

### Main Results– Fatty Acids, Cognition and Behavior

#### Demographic Information and Psychological Measures

There were no significant differences in reading ability, working memory, or parent and teacher rated behavior between children with blood data available (n = 493) and those without (n = 182) (Mann-Whitney U tests, see Table S3).

Examination of scores on the psychological measures for children grouped by age, socioeconomic status or sex did, however, reveal some significant associations. (For full results see Table S4). To summarize these:

- Reading ability varied significantly by age-group (p = <0.001), with older children having lower reading scores.
- Reading scores also varied by socioeconomic status (p = <0.003), with lower scores for children entitled to free school meals.
- Behavior problems (ADHD-type symptoms) were higher for children entitled to free school meals, according to both parent and teacher ratings (both p = <0.001).
- Teacher-rated ADHD symptomatology was higher in girls than boys (p = <0.007), although parent ratings did not differ by sex.
- Teacher ratings also varied slightly with age-group (p = <0.04), but without a linear age trend

### Fatty Acid Status and Cognitive Performance

Relationships between cognitive variables (age-standardized scores for reading and working memory) and blood fatty acids were investigated using non-parametric correlations (Spearman’s rho). Table 4 shows the results of these analyses.

**Table 4:** Fatty acid correlations with reading ability and working memory.

		Reading ability		Working memory	
		(n = 493)		(Digits forward, n = 493)	
		Rho†	P-Value	Rho†	P-Value
*Omega-3 fatty acids:*					
	**ALA** (18∶3, n-3)	0.07	0.147	−0.03	0.524
	**SDA** (20∶3, n-3)	−0.10	0.032	−0.10	0.035
	**EPA** (20∶5, n-3)	0.06	0.223	0.13	0.004
	**DPA** (22∶5, n-3)	0.10	0.032	0.09	0.038
	**DHA** (22∶6, n-3)	0.11	0.011	0.14	0.002
	**EPA+DHA** (“Omega-3Index”)	0.11	0.02	0.16	0.000
	**Total Omega-3**	0.11	0.014	0.13	0.003
*Omega-6 fatty acids:*					
	**LA** (18∶2, n-6)	0.08	0.081	0.02	0.649
	**GLA** (18∶3, n-6)	0.05	0.303	−0.06	0.224
	**DGLA** (20∶3, n-6)	0.04	0.44	0.01	0.815
	**AA** (20∶4, n-6)	0.07	0.15	0.04	0.392
	**Adrenic** (22∶4, n-6)	0.01	0.912	0.01	0.787
	**DPA** (22∶5, n-6)	−0.04	0.426	−0.03	0.579
	**Total Omega-6**	0.10	0.029	0.01	0.865

† Spearman’s correlations.

#### Reading ability

Reading scores were significantly and positively associated with the Omega-3 LC-PUFA DHA (p<0.003), DPA (p<0.04), EPA (p<0.005), and thus the EPA+DHA (“Omega-3 Index”) (p<0.001) as well as total Omega-3 (p<0.004), although a slight negative association was found with the shorter-chain Omega-3 SDA (p<0.04). Total Omega-6 fatty acids also showed a positive correlation with reading (p<0.03).

#### Working memory

Similarly, scores for Recall of Digits Forward (tapping auditory-verbal working memory) were significantly and positively associated with the Omega-3 LC-PUFA DHA (p<0.003), DPA (p<0.04), EPA (p<0.005), and thus the EPA+DHA (“Omega-3 Index”) (p<0.001) as well as total Omega-3 (p<0.004), although again, a slight negative association was found with the shorter-chain Omega-3 SDA (p<0.04). Scores for Recall of Digits Backward showed no significant associations with any fatty acid variables.

### Fatty Acid Status and Behavior

Table 5 summarizes the correlations found across all behavior (ADHD-type symptoms) rating scales (sub and global) for both parent and teacher ratings.

**Table 5:** Summary of result patterns for the correlation of fatty acids and the Conners’ Rating Scales.

		DHA	Omega-3 Total	EPA+DHA (“Omega-3 Index”)
		Rho† (p-value)	Rho† (p-value)	Rho† (p-value)
**PARENT Ratings:**				
**- Subscales**				
	Oppositional (n = 402)	−0.16 (0.001)	−0.13 (0.009)	−0.15 (0.003)
	Cognitive (n = 402)	−0.04 (0.404)	−0.05 (0.293)	−0.03 (0.536)
	Hyperactivity (n = 402)	−0.12 (0.012)	−0.13 (0.007)	−0.12 (0.019)
	Anxiety (n = 402)	−0.12 (0.018)	−0.05 (0.332)	−0.09 (0.065)
	Perfectionism (n = 401)	0.01 (0.847)	−0.01 (0.906)	0.00 (0.977)
	Social Problems (n = 402)	−0.03 (0.616)	−0.01 (0.778)	−0.03 (0.564)
	Psycho Somatic (n = 402)	−0.15 (0.002)	−0.13 (0.009)	−0.14 (0.006)
**- Global scales**				
	Conners’ ADHD Index (n = 402)	−0.09 (0.078)	−0.08 (0.128)	−0.06 (0.235)
	Global Restless-Impulsive (n = 401)	−0.11 (0.032)	−0.09 (0.081)	−0.08 (0.098)
	Global Emotional Lability (n = 401)	−0.17 (0.001)	−0.12 (0.013)	−0.17 (0.001)
	Global Total Index (n = 401)	−0.14 (0.007)	−0.10 (0.037)	−0.11 (0.022)
	DSM-IV Inattentive (n = 402)	−0.03 (0.528)	−0.02 (0.639)	−0.01 (0.780)
	DSM-IV Hyperactivity Impulsive (n = 402)	−0.13 (0.007)	−0.11 (0.024)	−0.11 (0.023)
	DSM-IV Total (n = 402)	−0.08 (0.096)	−0.07 (0.160)	−0.06 (0.204)
**TEACHER RATINGS:**				
- **Subscales**				
	Oppositional (n = 439)	−0.05 (0.288)	−0.04 (0.41)	−0.04 (0.422)
	Cognitive (n = 438)	0.02 (0.711)	0.04 (0.446)	0.03 (0.513)
	Hyperactivity (n = 433)	−0.01 (0.805)	−0.02 (0.727)	<0.01 (0.924)
	Anxiety (n = 439)	−0.10 (0.034)	−0.1 (0.028)	−0.09 (0.057)
	Perfectionism (n = 438)	0.04 (0.417)	0.03 (0.572)	0.04 (0.447)
	Social Problems (n = 438)	−0.09 (0.053)	−0.06 (0.21)	−0.09 (0.075)
**-Global scales**				
	Conners’ ADHD Index (n = 433)	0.02 (0.621)	0.02 (0.658)	0.04 (0.378)
	Global Restless-Impulsive (n = 438)	0.04 (0.428)	0.04 (0.394)	0.06 (0.191)
	Global Emotional Lability (n = 439)	−0.08 (0.098)	−0.03 (0.56)	−0.05 (0.297)
	Global Total Index (n = 438)	0.01 (0.873)	0.02 (0.619)	0.04 (0.463)
	DSM-IV Inattentive (n = 433)	0.02 (0.699)	0.03 (0.495)	0.03 (0.5)
	DSM-IV Hyperactivity Impulsive (n = 433)	−0.01 (0.796)	−0.02 (0.736)	<0.01 (0.996)
	DSM-IV Total (n = 433)	<0.01 (0.963)	0.01 (0.767)	0.02 (0.73)

† Spearman’s correlations.

For 9 out of 15 parent rating scales, DHA, total Omega-3, and the EPA+DHA (“Omega-3 Index”) were negatively and significantly related with parent-reported behavior problems. By contrast, prior to the adjusted analyses presented below, teacher ratings of behavior showed no significant associations with blood fatty acids. Except for an inverse relationship between anxiety and both DHA and Total Omega-3.

### Main Results– Fatty Acids, Cognition and Behavior Controlling for Demographics

In line with the study’s protocol, and following the earlier observation that some psychological variables did vary by demographics, their relationships with blood fatty acids were re-analyzed, adjusting for sex and SES (free school meal entitlement).

#### Cognition

Table 6 reports the regression coefficients for the relationships between blood fatty acids and cognition. The coefficients represent the observed change of the outcome variables for a 1% higher level of the respective fatty acid (or index).

**Table 6:** Adjusted relationship† between fatty acids, reading, and working memory.

	Reading ability			Working memory		
	(n = 493)			(Digits forward; n = 493)		
	Std. Coeff.††	Raw Coeff.	p-value	Std. Coeff.††	Raw Coeff.	p-value
*Omega-3 fatty acids:*						
** ALA** (18∶3, n-3)	0.03	1.37	0.459	−0.03	−1.01	0.462
** SDA** (20∶3, n-3)	−0.10	−16.62	0.022	−0.07	−9.15	0.089
** EPA** (20∶5, n-3)	0.04	1.99	0.398	0.13	5.26	0.240
** DPA** (22∶5, n-3)	0.08	3.10	0.081	0.07	2.09	0.114
** DHA** (22∶6, n-3)	0.09	1.81	0.042	0.14	2.12	0.130
** EPA+DHA** (“Omega-3 Index”)	0.09	1.38	0.056	0.15	1.90	0.040
** Total Omega-3**	0.10	1.22	0.034	0.13	1.25	0.310
*Omega-6 fatty acids:*						
** LA** (18∶2, n-6)	0.06	0.28	0.172	0.02	0.06	0.676
** GLA** (18∶3, n-6)	0.07	2.95	0.148	−0.04	−1.29	0.393
** DGLA** (20∶3, n-6)	−0.50	−0.17	0.905	−0.02	−0.54	0.606
** AA** (20∶4, n-6)	0.05	0.39	0.281	0.02	0.13	0.632
** Adrenic** (22∶4, n-6)	−0.02	−0.86	0.693	−0.02	−0.54	0.738
** DPA** (22∶5, n-6)	−0.03	−2.88	0.551	−0.05	−4.21	0.239
** Total Omega-6**	0.06	0.20	0.155	0.01	0.03	0.747

† OLS regression controlling for Gender and SES.

†† Beta-coefficient were standardized using the ratio of standard deviations (sd_x_/sd_y_).

The results indicate that the association found between reading ability and DHA is robust. Similarly, the relationships between working memory (forward) and DHA as well as EPA hold when controlling for children’s sex and socio-economic status. The weakly negative association of SDA and reading ability was also still significant after the adjustment. No associations were found between these cognitive variables and any Omega-6 fatty acids.

#### Behavior

Re-analyzing the parent-rated behavior with adjustment for sex and SES yielded the following significant relationships:

- Oppositional sub-scale (DHA: Std. Coeff. −0.175, Raw Coeff. −3.865, p<0.000; EPA+DHA: −0.151, −2.661, p<0.002);
- Global Emotional Lability scale (DHA: −0.178, −3.541, p<0.000; EPA+DHA −0.168, −2.678, p<0.001)
- Anxiety (DHA −0.123, −2.258, p<0.014)
- Psychosomatic symptoms (DHA −0.116, −2.151, p<0.020)
- Conners’ Global Index (DHA −0.122, −2.388, p<0.013)

All were significantly and inversely related with DHA and/or EPA+DHA (“Omega-3 Index”), as indicated above.

## Discussion

In this large sample of healthy UK school children thought to be underperforming in reading, average blood Omega-3 LC-PUFA concentrations (EPA+DHA) were 2.46%, which is well below the minimum recommended for good cardiovascular health in adults. Similar findings appear to be emerging from the first large-scale studies of blood fatty acid profiles in children from other European countries, which - like our study - have used capillary whole blood collected from fingerstick samples [26]. They are also in line with findings from a venous blood study in German infants [27].

Concentrations below 4% EPA+DHA in red cell membranes (i.e. the Omega-3 index) are considered to signify high cardiovascular risk, and 8–12% the optimal range [22]. The longer term implications of the very low values found in these UK schoolchildren obviously cannot be known, but give cause for concern.

With respect to mental health and development, blood Omega-3 LC-PUFA status in these UK children significantly predicted both their behavior and their cognitive performance. Specifically – higher levels of Omega-3 LC-PUFA, and DHA in particular, were associated with better reading and working memory performance, and fewer ADHD-type symptoms, even when controlling for sex and socioeconomic status. These results are particularly noteworthy given the somewhat restricted range of both reading ability and blood Omega-3 LC-PUFA in this sample. Unsurprisingly, lower reported fish intakes in our sample were associated with lower blood concentrations of Omega-3 LC-PUFA. Parent reports showed that almost 9 out of 10 children failed to meet current UK dietary guidelines which recommend 2 portions of fish per week [28]. The association between fish intake and blood Omega-3 LC-PUFA is a limitation of the study, i.e. to what extent are other nutrients found in fish predictors of better reading ability and cognition. Investigating these potentially confounding relationships should be considered in future intervention studies.

Generalisability to all children aged 7 to 9 years attending English primary schools is obviously problematic, as this sample was selected for presumed underperformance in reading (below average based on national attainment tests carried out at age 7 years and/or teachers’ judgments). This was necessary in order to fulfill the screening requirements of a subsequent intervention trial (The DOLAB study) [17]. Nonetheless, our formal testing showed that the actual distribution of reading scores in this sample was within the normal population range. Further studies sampling the full range of reading ability would be needed to confirm the associations we have shown here between Omega-3 DHA and reading, working memory and behavior, but these findings are likely to be generalizable to similar healthy, mainstream schoolchildren.

The percentage of children eligible for free school meals was slightly lower than national figures (15.6% versus 18.6%); and the proportion of non-white children was substantially lower (8.1% versus 22.3%). This difference in ethnicity almost certainly reflected our inclusion criterion requiring that children use English as their first language at home. However, it means that generalizability of these findings to ethnic minority groups cannot be assumed, particularly given that some important genetic influences on LC-PUFA metabolism and status are known to vary with ancestry [29].

The percentage of boys in the present sample (55.2%) contrasts a little with 51% in the national sample, almost certainly reflecting the fact that boys in this age group are more likely than girls to be identified as underperforming in reading. However, our analyses controlled for any sex differences, and these were not evident in most of the relationships of interest. The sex ratio in this study was much closer to national averages than most previous studies of Omega-3 LC-PUFA in relation to child behavior and learning. This is because most have involved children with developmental conditions such as ADHD, which are more commonly diagnosed in boys.

In this study, low Omega-3 LC-PUFA status was even more pronounced in girls than boys. Sex differences in fatty acid metabolism have been well-documented in adults and typically LC-PUFA status in women is higher [30]. However, these metabolic differences primarily reflect hormonal influences on the synthesis of LC-PUFA, hence they are most evident only in women of child-bearing age. Possible sex differences merit consideration in future studies.

Major strengths of this study include the large sample size and the use of objective measures of blood Omega-3 status. Most previous studies seeking to investigate links with psychological functioning at the general population level have relied on either dietary or economic data – both of which are unreliable [23]. A further strength is the use of well-validated, age-standardized tests of behavior and cognition which also have real practical relevance.

The finding that low-Omega-3 LC-PUFA, and DHA in particular, predict behavior and learning problems in this large sample of healthy, but underperforming children attending mainstream schools suggests that the benefits from dietary supplementation found in ADHD and related conditions may extend to a wider population [10]. This question can only be addressed by well-powered intervention studies, but meanwhile, the low blood Omega-3 status found across this sample would indicate that an increased dietary intake might be beneficial on general health grounds.

## Supporting Information

Materials S1 Further information on those for whom blood data were available.
(DOCX)

Materials S2 Methods for capillary whole blood fatty acid analysis.
(DOCX)

Table S1 Mean levels of Conners’ subscales* in overall sample.
(DOCX)

Table S2 Blood fatty acid levels by pupils’ gender, age, and free school meal entitlement.
(DOCX)

Table S3 Outcome variables (Comparison between pupils with and without blood data).
(DOCX)

Table S4 Demographic variables and outcome variables.
(DOCX)

## References

[1] Ryan AS, Astwood JD, Gautier S, Kuratko CN, Nelson EB, et al (2010) Effects of long-chain polyunsaturated fatty acid supplementation on neurodevelopment in childhood: a review of human studies. Prostaglandins Leukot Essent Fatty Acids 82: 305–314. 20188533 10.1016/j.plefa.2010.02.007

[2] Schuchardt JP, Huss M, Stauss-Grabo M, Hahn A (2010) Significance of long-chain polyunsaturated fatty acids (PUFAs) for the development and behaviour of children. Eur J Pediatr 169: 149–164. 19672626 10.1007/s00431-009-1035-8

[3] Blasbalg TL, Hibbeln JR, Ramsden CE, Majchrzak SF, Rawlings RR (2011) Changes in consumption of omega-3 and omega-6 fatty acids in the United States during the 20th century. Am J Clin Nutr 93: 950–962. 21367944 10.3945/ajcn.110.006643 PMC3076650

[4] Riediger ND, Othman RA, Suh M, Moghadasian MH (2009) A systemic review of the roles of n-3 fatty acids in health and disease. Journal of the American Dietetic Association 109: 668–679. 19328262 10.1016/j.jada.2008.12.022

[5] Ramakrishnan U, Imhoff-Kunsch B, DiGirolamo AM (2009) Role of docosahexaenoic acid in maternal and child mental health. American Journal of Clinical Nutrition 89: 958–962. 10.3945/ajcn.2008.26692F PMC2667651 19176728

[6] Richardson AJ (2006) Omega-3 fatty acids in ADHD and related neurodevelopmental disorders. Int Rev Psychiatry 18: 155–172. 16777670 10.1080/09540260600583031

[7] Stevens LJ, Zentall SS, Deck JL, Abate ML, Watkins BA, et al (1995) Essential fatty acid metabolism in boys with attention-deficit hyperactivity disorder. Am J Clin Nutr 62: 761–768. 7572706 10.1093/ajcn/62.4.761

[8] Chen JR, Hsu SF, Hsu CD, Hwang LH, Yang SC (2004) Dietary patterns and blood fatty acid composition in children with attention-deficit hyperactivity disorder in Taiwan. J Nutr Biochem 15: 467–472. 15302081 10.1016/j.jnutbio.2004.01.008

[9] Sinn N, Bryan J, Wilson C (2008) Cognitive effects of polyunsaturated fatty acids in children with attention deficit hyperactivity disorder symptoms: a randomised controlled trial. Prostaglandins Leukot Essent Fatty Acids 78: 311–326. 18514501 10.1016/j.plefa.2008.04.004

[10] Bloch MH, Qawasmi A (2011) Omega-3 Fatty Acid Supplementation for the Treatment of Children With Attention-Deficit/Hyperactivity Disorder Symptomatology: Systematic Review and Meta-Analysis. Journal of the American Academy of Child and Adolescent Psychiatry 50: 991–1000. 21961774 10.1016/j.jaac.2011.06.008 PMC3625948

[11] Richardson AJ, Montgomery P (2005) The Oxford-Durham study: a randomized, controlled trial of dietary supplementation with fatty acids in children with developmental coordination disorder. Pediatrics 115: 1360–1366. 15867048 10.1542/peds.2004-2164

[12] Burgess JR, Stevens LJ (2003) Essential fatty acids in relation to attention deficit hyperactivity disorder. In: Glen I, Horrobin DF, editors. Phospholipid spectrum disorders in psychiatry and neurology Carnforth: Marius Press. 511–519.

[13] Cyhlarova E, Bell JG, Dick JR, Mackinlay EE, Stein JF, et al (2007) Membrane fatty acids, reading and spelling in dyslexic and non-dyslexic adults. Eur Neuropsychopharmacol 17: 116–121. 16997534 10.1016/j.euroneuro.2006.07.003

[14] Kirby A, Woodward A, Jackson S, Wang Y, Crawford MA (2010) A double-blind, placebo-controlled study investigating the effects of omega-3 supplementation in children aged 8–10 years from a mainstream school population. Res Dev Disabil 31: 718–730. 20171055 10.1016/j.ridd.2010.01.014

[15] Bailey-Hall E, Nelson EB, Ryan AS (2008) Validation of a rapid measure of blood PUFA levels in humans. Lipids 43: 181–186. 18084784 10.1007/s11745-007-3140-7

[16] Marangoni F, Colombo C, Galli C (2004) A method for the direct evaluation of the fatty acid status in a drop of blood from a fingertip in humans: applicability to nutritional and epidemiological studies. Anal Biochem 326: 267–272. 15003567 10.1016/j.ab.2003.12.016

[17] Richardson AJ, Burton JR, Sewell RP, Spreckelsen TF, Montgomery P (2012) Docosahexaenoic Acid for Reading, Cognition and Behavior in Children Aged 7–9 Years: A Randomized, Controlled Trial (The DOLAB Study). PLoS one 7: e43909 doi:43910.41371/journal.pone.0043909 22970149 10.1371/journal.pone.0043909 PMC3435388

[18] Department of Education (2001) Special Educational Needs Code of Practice - DfES/581/2001. Available online: https://www.education.gov.uk/publications/eOrderingDownload/DfES%200581%20200MIG2228.pdf. Accessed: 2013 Mar 20.

[19] Standards and Testing Agency (2012) National Curriculum assessments - Assessment and reporting arrangements Key Stage 1. Available online: http://www.education.gov.uk/schools/teachingandlearning/assessment/a00197251/assessment-and-reporting-arrangements. Accessed: 2012 May 15.

[20] Hobbs G, Vignoles A (2010) Is children’s free school meal ‘eligibility’ a good proxy for family income? British Educational Research Journal 36: 673–690.

[21] Morrison WR, Smith LM (1964) Preparation of Fatty Acid Methyl Esters+Dimethylacetals from Lipids with Boron Fluoride-Methanol. Journal of Lipid Research 5: 600–608. 14221106

[22] Harris WS, von Schacky C (2004) The Omega-3 Index: a new risk factor for death from coronary heart disease? Preventive Medicine 39: 212–220. 15208005 10.1016/j.ypmed.2004.02.030

[23] Harris WS, Klurfeld DM (2011) Twentieth-century trends in essential fatty acid intakes and the predicted omega-3 index: evidence versus estimates. Am J Clin Nutr 93: 907–908. 21430117 10.3945/ajcn.111.014365

[24] Elliott CD, Smith P, McCulloch K (1997) British Ability Scales II: Technical Manual; Wales NFfERiEa, editor. London: NferNelson.

[25] Conners KC (1997) Conners’ Rating Scales-Revised – Instruments for use with Children and Adolescents: Technical Manual. New York: Multi-Health Systems.

[26] Galli C, Risé C, Ghezzi S, Colombo C, Ahrens W, et al.. (2010) Blood levels of n-3 long chain polyunsaturated fatty acids differ significantly in children from 8 European countries: the IDEFICS study. Poster 9th Conference of the International Society for the Study of Fatty Acids and Lipids (ISSFAL): Maastricht, 2010 May 31.

[27] Glaser C, Demmelmair H, Sausenthaler S, Herbarth O, Heinrich J, et al.. (2010) Fatty acid composition of serum glycerophospholipids in children. J Pediatr 157: 826–831 e821. 10.1016/j.jpeds.2010.05.001 20646712

[28] Food Standards Agency (2010) Eat Well - Your guide to healthy eating (2010 amended edition). Available online: http://www.food.gov.uk/multimedia/pdfs/publication/eatwell0708.pdf. Accessed: 2013 March 20.

[29] Mathias RA, Sergeant S, Ruczinski I, Torgerson DG, Hugenschmidt CE, et al. (2011) The impact of FADS genetic variants on omega-6 polyunsaturated fatty acid metabolism in African Americans. Bmc Genetics 12: doi:–––10.1186/1471–2156–1112–1150 10.1186/1471-2156-12-50 PMC3118962 21599946

[30] Decsi T, Kennedy K (2011) Sex-specific differences in essential fatty acid metabolism. American Journal of Clinical Nutrition 94: 1914–1919. 10.3945/ajcn.110.000893 22089435

