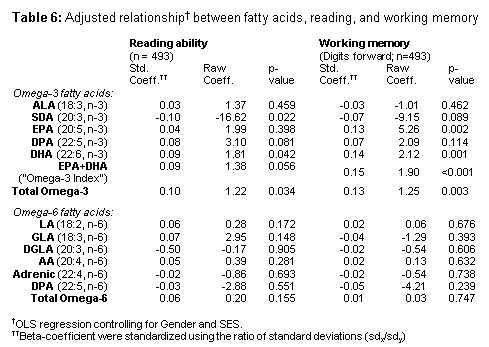# Correction: Low Blood Long Chain Omega-3 Fatty Acids in UK Children Are Associated with Poor Cognitive Performance and Behavior: A Cross-Sectional Analysis from the DOLAB Study

**DOI:** 10.1371/annotation/26c6b13f-b83a-4a3f-978a-c09d8ccf1ae2

**Published:** 2013-09-06

**Authors:** Paul Montgomery, Jennifer R. Burton, Richard P. Sewell, Thees F. Spreckelsen, Alexandra J. Richardson

There was an error in the Funding statement. The correct Funding statement is: The study was funded by Martek Biosciences Inc. (http://www.lifesdha.com/), and The Waterloo Foundation for its support for Dr Richardson's work in this area. The funders had no role in study design, data collection and analysis, decision to publish, or preparation of the manuscript.

There was an error in Table 6. The correct version of Table 6 is available here: 

**Figure:**