# Supplementary material for: Low Blood Long Chain Omega-3 Fatty Acids in UK Children Are Associated with Poor Cognitive Performance and Behavior: A Cross-Sectional Analysis from the DOLAB Study
Source: PLoS One. 2013 Jun 24;8(6):e66697. doi: 10.1371/journal.pone.0066697 (PMC3691187; doi:10.1371/journal.pone.0066697)
Supplement: Materials S1 — Further information on those for whom blood data were available. (DOCX) [file pone.0066697.s001.docx]

**Materials S1: Further information on those for whom blood data were available.**

***Vitamins and supplements***

The parents of 62/431 (14.4%) of children reported that their children currently took vitamins or supplements regularly, however only 20 (4.6%) of these children were currently taking an Omega 3 supplement.

***Medication***

82/440 (18.6%) of children’s parents reported that their child currently took medication. The majority, 61 (74%) of these children were taking medication for an atopic condition (i.e. asthma, eczema or hay fever). Other chronic conditions requiring medication were constipation, diabetes and a thyroid condition.
