# Supplementary material for: Low Blood Long Chain Omega-3 Fatty Acids in UK Children Are Associated with Poor Cognitive Performance and Behavior: A Cross-Sectional Analysis from the DOLAB Study
Source: PLoS One. 2013 Jun 24;8(6):e66697. doi: 10.1371/journal.pone.0066697 (PMC3691187; doi:10.1371/journal.pone.0066697)
Supplement: Materials S2 — Methods for capillary whole blood fatty acid analysis. (DOCX) [file pone.0066697.s002.docx]

**Materials S2: Methods for capillary whole blood fatty acid analysis**

Capillary whole blood from a finger stick was analyzed for total lipid fatty acids. A small lancet device (BD Microtainer Contact-Activated Lancets or similar, Emergency Medical Products) was applied to the subject’s finger and drops of blood were collected on a 1.5 x 1.5 cm piece of filter paper (Whatman 3MM chromatography paper, Whatman Inc.) impregnated with BHT and prepared according to the methods of Ichihara et al. [1]. The sample was dried at room temperature overnight and analyzed within 7 days of the blood collection. The blood saturated filter paper samples were directly methylated without a prior extraction step. Tricosanoic free fatty acid (23:0) (NuCheck Prep Elysian, MN, U.S.A) was added to each sample as an internal standard. The total lipids were saponified with 0.5 N methanolic sodium hydroxide and the fatty acids were converted to methyl esters with 14% BF_3_/methanol (Sigma) at 100°C for 30 minutes (Morrison and Smith, 1964 [2]). The methyl esters were extracted with hexane and washed with saturated sodium chloride distilled water. Butylated hydroxytoluene was added before saponification and all samples were purged with N_2_ throughout the process to minimize oxidation. Fatty acid methyl esters were analyzed by gas-liquid-chromatography using a Hewlett Packard 6890 detector equipped with a flame ionization. The fatty acid methyl esters were separated on a 30 meter FAMEWAX capillary column (Restek, Bellefonte, PA; 0.25 mm diameter, 0.25 μm coating thickness) using hydrogen at a flow rate of 2.1 mL/min with a split ratio of 20:1. The chromatographic run parameters included an oven starting temperature of 130°C that was increased at 6°C/min to 225°C, where it was held for 20 minutes before increasing to 250°C at 15°C/min, with a final hold of 5 minutes. The injector and detector temperatures were constant at 220°C and 230°C respectively. Peaks were identified by comparison of retention times with external fatty acid methyl ester standard mixtures from NuCheck Prep (Elysian, MN, U.S.A). The fatty acid profiles were expressed as a percent of the total μg of fatty acid (weight percent).

[1] [Ichihar K](http://www.ncbi.nlm.nih.gov/pubmed?term=Ichihar%20K%5BAuthor%5D&cauthor=true&cauthor_uid=12056596), [Waku K](http://www.ncbi.nlm.nih.gov/pubmed?term=Waku%20K%5BAuthor%5D&cauthor=true&cauthor_uid=12056596), [Yamaguchi C](http://www.ncbi.nlm.nih.gov/pubmed?term=Yamaguchi%20C%5BAuthor%5D&cauthor=true&cauthor_uid=12056596), [Saito K](http://www.ncbi.nlm.nih.gov/pubmed?term=Saito%20K%5BAuthor%5D&cauthor=true&cauthor_uid=12056596), [Shibahara A](http://www.ncbi.nlm.nih.gov/pubmed?term=Shibahara%20A%5BAuthor%5D&cauthor=true&cauthor_uid=12056596), [Miyatani S](http://www.ncbi.nlm.nih.gov/pubmed?term=Miyatani%20S%5BAuthor%5D&cauthor=true&cauthor_uid=12056596), [Yamamoto K](http://www.ncbi.nlm.nih.gov/pubmed?term=Yamamoto%20K%5BAuthor%5D&cauthor=true&cauthor_uid=12056596). (2002) A Convenient Method for Determination of C20-22 PUFA Composition of Glycerolipids in Blood and Breast Milk. Lipids 37:523-526.

[2] Morrison, WR.; Smith LM. (1964) Preparation of fatty acid methyl esters and dimethylacetals from lipids with boron fluoride–methanol. J. Lipid Res. 5: 600-608.
