# Supplementary material for: Low Blood Long Chain Omega-3 Fatty Acids in UK Children Are Associated with Poor Cognitive Performance and Behavior: A Cross-Sectional Analysis from the DOLAB Study
Source: PLoS One. 2013 Jun 24;8(6):e66697. doi: 10.1371/journal.pone.0066697 (PMC3691187; doi:10.1371/journal.pone.0066697)
Supplement: Table S1 — Mean levels of Conners’ subscales* in overall sample. (DOCX) [file pone.0066697.s003.docx]

|  | | | | |
| --- | --- | --- | --- | --- |
| **Table S1: Mean levels of Conners’ subscales* in overall sample.** | | | | |
|  |  | **N** | **Mean** | **Sd.** |
| **Parents' Rating Subscales:** | |  |  |  |
|  | Oppositional | 402 | 55.13 | 11.75 |
|  | Cognitive | 402 | 57.89 | 11.30 |
|  | Hyperactivity | 402 | 53.34 | 9.47 |
|  | Anxiety | 402 | 50.55 | 9.83 |
|  | Perfectionism | 401 | 48.09 | 8.89 |
|  | Social Problems | 402 | 54.60 | 11.37 |
|  | Psycho-Somatic | 402 | 54.17 | 12.36 |
|  | ADHD Index | 402 | 56.52 | 11.03 |
|  | Global Restless-Impulsive | 401 | 55.42 | 10.30 |
|  | Global Index Emotional Lability | 401 | 52.51 | 10.62 |
|  | Global Index Total Index | 401 | 54.99 | 10.43 |
|  | DSM-IV Inattentive | 402 | 55.35 | 11.08 |
|  | DSM-IV Hyperactivity-Impulsive | 402 | 55.69 | 10.92 |
|  | DSM-IV Total | 402 | 55.90 | 10.71 |
|  |  |  |  |  |
| **Teachers' Rating Subscales:** | |  |  |  |
|  | Oppositional | 439 | 54.53 | 12.62 |
|  | Cognitive | 438 | 60.25 | 9.62 |
|  | Hyperactivity | 433 | 53.40 | 10.90 |
|  | Anxiety | 439 | 56.80 | 12.20 |
|  | Perfectionism | 438 | 48.25 | 8.15 |
|  | Social Problems | 438 | 53.84 | 11.16 |
|  | ADHD Index | 433 | 55.83 | 11.30 |
|  | Global Restless-Impulsive | 438 | 56.07 | 11.64 |
|  | Global Index Emotional Lability | 439 | 52.85 | 11.96 |
|  | Global Index Total Index | 438 | 55.60 | 11.69 |
|  | DSM-IV Inattentive | 433 | 57.53 | 10.33 |
|  | DSM-IV Hyperactivity-Impulsive | 433 | 52.42 | 11.05 |
|  | DSM-IV Total | 433 | 55.84 | 10.31 |
|  | | | | |

*Conner’s Rating Scales – Long version.
