# Supplementary material for: Low Blood Long Chain Omega-3 Fatty Acids in UK Children Are Associated with Poor Cognitive Performance and Behavior: A Cross-Sectional Analysis from the DOLAB Study
Source: PLoS One. 2013 Jun 24;8(6):e66697. doi: 10.1371/journal.pone.0066697 (PMC3691187; doi:10.1371/journal.pone.0066697)
Supplement: Table S2 — Blood fatty acid levels by pupils’ gender, age, and free school meal entitlement. (DOCX) [file pone.0066697.s004.docx]

| **Table S2:** **Blood fatty acid levels by pupils’ gender, age, and free school meal entitlement** | | | | | | | | | | | | | | | | | | |
| --- | --- | --- | --- | --- | --- | --- | --- | --- | --- | --- | --- | --- | --- | --- | --- | --- | --- | --- |
|  |  | **Total** |  | **Gender** | |  |  |  | **Age (7, 8, 9 years of age)** | | |  |  |  | **SES (Free meals)** | |  |  |
|  | **N** | Mean  (sd) |  | Male  (sd) | Female  (sd) |  | **z**  **(p-value)*** |  | 6/7 years  (sd) | 8 years  (sd) | 9/10 years  (sd) |  | **chi2**  **(p-value)**** |  | Freemeals  (sd) | No freemeals  (sd) |  | **z**  **(p-value)*** |
| Omega-3 |  |  |  |  |  |  |  |  |  |  |  |  |  |  |  |  |  |  |
| **ALA (18:3, n-3)** | **493** | 0.54 (0.254) |  | 0.55 (0.255) | 0.54 (0.252) |  | 0.187 (0.852) |  | 0.55 (0.31) | 0.53 (0.212) | 0.55 (0.246) |  | 0.492 (0.782) |  | 0.52 (0.323) | 0.55 (0.239) |  | 1.68 (0.093) |
| **SDA (20:3, n-3)** | **493** | 0.05 (0.065) |  | 0.05 (0.063) | 0.04 (0.066) |  | -0.594 (0.552) |  | 0.04 (0.056) | 0.04 (0.052) | 0.05 (0.08) |  | 0.106 (0.948) |  | 0.04 (0.06) | 0.05 (0.066) |  | -0.092 (0.927) |
| **EPA (20:5, n-3)** | **493** | 0.56 (0.2) |  | 0.57 (0.218) | 0.55 (0.175) |  | -0.296 (0.768) |  | 0.57 (0.245) | 0.55 (0.174) | 0.56 (0.186) |  | 1.323 (0.516) |  | 0.56 (0.183) | 0.56 (0.203) |  | -0.266 (0.79) |
| **DPA (22:5, n-3)** | **493** | 1.03 (0.266) |  | 1.05 (0.185) | 1 (0.339) |  | -4.113 (>0.001) |  | 1.06 (0.218) | 1.02 (0.311) | 1.01 (0.25) |  | 7.637 (0.022) |  | 1.08 (0.408) | 1.02 (0.23) |  | -0.57 (0.569) |
| **DHA (22:6, n-3)** | **493** | 1.9 (0.53) |  | 1.95 (0.53) | 1.85 (0.525) |  | -2.1 (0.036) |  | 1.97 (0.568) | 1.87 (0.471) | 1.88 (0.551) |  | 2.671 (0.263) |  | 1.82 (0.454) | 1.92 (0.541) |  | 1.072 (0.284) |
| **EPA+DHA**  **(“Omega-3 Index”)** | **493** | 2.46 (0.651) |  | 2.51 (0.658) | 2.4 (0.639) |  | -1.83 (0.067) |  | 2.55 (0.734) | 2.42 (0.55) | 2.45 (0.673) |  | 1.983 (0.371) |  | 2.39 (0.532) | 2.48 (0.671) |  | 0.706 (0.48) |
| **Total Omega-3** | **493** | 4.08 (0.823) |  | 4.16 (0.813) | 3.99 (0.828) |  | -2.183 (0.029) |  | 4.2 (0.935) | 4.01 (0.731) | 4.06 (0.813) |  | 3.757 (0.153) |  | 4.03 (0.766) | 4.09 (0.834) |  | 0.88 (0.379) |
| Omega-6 |  |  |  |  |  |  |  |  |  |  |  |  |  |  |  |  |  |  |
| **LA (18:2, n-6)** | **493** | 19.19 (2.294) |  | 19.19 (2.356) | 19.19 (2.22) |  | 0.007 (0.994) |  | 19.73 (2.064) | 19.12 (2.378) | 18.87 (2.314) |  | 12.298 (0.002) |  | 19.45 (2.128) | 19.15 (2.322) |  | -1.025 (0.305) |
| **GLA (18:3, n-6)** | **493** | 0.31 (0.233) |  | 0.34 (0.243) | 0.28 (0.217) |  | -2.98 (0.003) |  | 0.31 (0.228) | 0.33 (0.251) | 0.30 (0.22) |  | 0.367 (0.832) |  | 0.28 (0.22) | 0.32 (0.235) |  | 1.875 (0.061) |
| **DGLA (20:3, n-6)** | **493** | 1.56 (0.341) |  | 1.62 (0.34) | 1.48 (0.327) |  | -4.394 (>0.001) |  | 1.55 (0.312) | 1.56 (0.352) | 1.56 (0.352) |  | 0.004 (0.998) |  | 1.5 (0.287) | 1.57 (0.349) |  | 1.455 (0.146) |
| **AA (20:4, n-6)** | **493** | 8.17 (1.31) |  | 8.35 (1.262) | 7.94 (1.335) |  | -3.701 (>0.001) |  | 8.50 (1.329) | 8.1 (1.224) | 7.98 (1.337) |  | 10.915 (0.004) |  | 8.17 (1.395) | 8.17 (1.296) |  | >0.001 (1.000) |
| **Adrenic (22:4, n-6)** | **493** | 1.11 (0.221) |  | 1.14 (0.215) | 1.07 (0.221) |  | -3.787 (>0.001) |  | 1.12 (0.223) | 1.11 (0.221) | 1.10 (0.219) |  | 0.634 (0.728) |  | 1.12 (0.24) | 1.10 (0.217) |  | -0.426 (0.670) |
| **DPA (22:5, n-6)** | **493** | 0.25 (0.100) |  | 0.27 (0.100) | 0.24 (0.096) |  | -5.019 (>0.001) |  | 0.26 (0.097) | 0.26 (0.1) | 0.25 (0.102) |  | 0.106 (0.948) |  | 0.27 (0.072) | 0.25 (0.104) |  | -1.47 (0.142) |
| **Total Omega-6** | **493** | 30.84 (3.34) |  | 31.17 (3.397) | 30.44 (3.23) |  | -2.368 (0.018) |  | 31.71 (3.1) | 30.71 (3.398) | 30.32 (3.348) |  | 15.17 (0.001) |  | 31.06 (3.106) | 30.8 (3.384) |  | -0.201 (0.841) |
|  |  |  |  |  |  |  |  |  |  |  |  |  |  |  |  |  |  |  |
|  | | | | | | | | | | | | | | | | | | |

* Mann-Whitney Test

**Kruskal-Wallis Test
