# Supplementary material for: Low Blood Long Chain Omega-3 Fatty Acids in UK Children Are Associated with Poor Cognitive Performance and Behavior: A Cross-Sectional Analysis from the DOLAB Study
Source: PLoS One. 2013 Jun 24;8(6):e66697. doi: 10.1371/journal.pone.0066697 (PMC3691187; doi:10.1371/journal.pone.0066697)
Supplement: Table S3 — Outcome variables (Comparison between pupils with and without blood data). (DOCX) [file pone.0066697.s005.docx]

| **Table S3: Outcome variables (Comparison between pupils with and without blood data).** | | | | | | | | | | |
| --- | --- | --- | --- | --- | --- | --- | --- | --- | --- | --- |
| **Outcome Variables** | **Total**  **Sample** | |  | **Blood data**  **available** | |  | **No blood**  **data** | |  | **Mann-Whitney Test** |
|  | N | mean (sd.) |  | N | mean (sd.) |  | N | mean (sd.) |  | z (p-value) |
| **BAS Reading**  **Ability** | 675 | 90.58 (10.55) |  | 493 | 90.91 (10.55) |  | 182 | 89.7 (10.53) |  | 1.306 (0.19) |
| **BAS Working**  **Memory**  (Digits forward) | 675 | 41.57 (8.02) |  | 493 | 41.48 (7.76) |  | 182 | 41.82 (8.72) |  | -0.729 (0.47) |
| **CPRS-L**  **DSM Global**  (parent) | 536 | 55.33 (10.92) |  | 401 | 54.99 (10.43) |  | 135 | 56.36 (12.25) |  | -0.667 (0.51) |
| **CTRS-L**  **DSM Global**  (teacher) | 602 | 55.19 (11.41) |  | 438 | 55.6 (11.69) |  | 164 | 54.1 (10.57) |  | 1.243 (0.21) |
|  | | | | | | | | | | |
