# Supplementary material for: Low Blood Long Chain Omega-3 Fatty Acids in UK Children Are Associated with Poor Cognitive Performance and Behavior: A Cross-Sectional Analysis from the DOLAB Study
Source: PLoS One. 2013 Jun 24;8(6):e66697. doi: 10.1371/journal.pone.0066697 (PMC3691187; doi:10.1371/journal.pone.0066697)
Supplement: Table S4 — Demographic variables and outcome variables. (DOCX) [file pone.0066697.s006.docx]

| **Table S4: Demographic variables and outcome variables.** | | | | | | | |
| --- | --- | --- | --- | --- | --- | --- | --- |
| **Gender:** | | |  | **BAS Reading Ability** | **BAS Working Memory (**Digits forward**)** | **CPRS-L DSM Global**  (parent) | **CTRS-L DSM Global**  (teacher) |
|  | Male | | |  |  |  |  |
|  |  | N | | 376 | 376 | 302 | 340 |
|  |  | Mean(sd.) | | 91.07 (11.21) | 41.84 (7.98) | 54.88 (10.67) | 53.91 (10.41) |
|  | Female | | |  |  |  |  |
|  |  | N | | 299 | 299 | 234 | 262 |
|  |  | Mean(sd.) | | 89.97 (9.64) | 41.24 (8.07) | 55.91 (11.23) | 56.85 (12.41) |
|  |  |  | |  |  |  |  |
|  | **z (p-value)*** | | | **-1.15 (0.252)** | **-0.59 (0.553)** | **0.97 (0.331)** | **2.69 (0.007)** |
| **Age**: | |  | |  |  |  |  |
|  | 9-10 years | | |  |  |  |  |
|  |  | N | | 244 | 244 | 188 | 225 |
|  |  | Mean(sd.) | | 89.31 (10.79) | 42.04 (8.21) | 55.36 (10.98) | 56.44 (11.45) |
|  | 8 years | | |  |  |  |  |
|  |  | N | | 248 | 248 | 198 | 219 |
|  |  | Mean(sd.) | | 89.54 (10.24) | 40.73 (8.13) | 55.53 (10.36) | 53.97 (10.96) |
|  | 6-7years | | |  |  |  |  |
|  |  | N | | 183 | 183 | 150 | 158 |
|  |  | Mean(sd.) | | 93.7 (10.06) | 42.1 (7.55) | 55.03 (11.62) | 55.11 (11.82) |
|  |  |  | |  |  |  |  |
|  | **chi2 (p-value)**** | | | **23.66 (0)** | **3.69 (0.158)** | **0.97 (0.617)** | **6.49 (0.039)** |
| **Free School meals:** | | | |  |  |  |  |
|  | Yes |  | |  |  |  |  |
|  |  | N | | 117 | 117 | 76 | 107 |
|  |  | Mean(sd.) | | 87.91 (10.57) | 41.28 (8.2) | 59.29 (11.63) | 58.69 (12.39) |
|  | No |  | |  |  |  |  |
|  |  | N | | 558 | 558 | 460 | 495 |
|  |  | Mean(sd.) | | 91.15 (10.47) | 41.64 (7.99) | 54.68 (10.67) | 54.43 (11.05) |
|  |  |  | |  |  |  |  |
|  | **z (p-value)*** | | | **3.02 (0.003)** | **0.51 (0.613)** | **-3.37 (0.001)** | **-3.33 (0.001)** |
| **Urban vs. Rural** | | | |  |  |  |  |
|  | Rural | | |  |  |  |  |
|  |  | N | | 237 | 237 | 200 | 224 |
|  |  | Mean(sd.) | | 91.93 (11.1) | 42.36 (8.22) | 54.96 (10.39) | 55.48 (12.03) |
|  | Urban | | |  |  |  |  |
|  |  | N | | 438 | 438 | 336 | 378 |
|  |  | Mean(sd.) | | 89.86 (10.18) | 41.15 (7.89) | 55.55 (11.24) | 55.02 (11.03) |
|  |  |  | |  |  |  |  |
|  | **z (p-value)*** | | | **-2.36 (0.018)** | **-1.66 (0.097)** | **0.28 (0.781)** | **-0.19 (0.849)** |
|  | | | | | | | |

*Mann-Whitney Test

**Kruskal-Wallis test (H0 all groups are the same).
